# Lung and Gut Microbiota as Potential Hidden Driver of Immunotherapy Efficacy in Lung Cancer

**DOI:** 10.1155/2019/7652014

**Published:** 2019-11-11

**Authors:** Carmine Carbone, Geny Piro, Vincenzo Di Noia, Ettore D'Argento, Emanuele Vita, Miriam Grazia Ferrara, Sara Pilotto, Michele Milella, Giovanni Cammarota, Antonio Gasbarrini, Giampaolo Tortora, Emilio Bria

**Affiliations:** ^1^Comprehensive Cancer Center, Fondazione Policlinico Universitario Agostino Gemelli, IRCCS, Rome, Italy; ^2^Università Cattolica del Sacro Cuore, Rome, Italy; ^3^Section of Medical Oncology, Department of Medicine, University of Verona and University Hospital Trust, Verona, Italy; ^4^Digestive Disease Center, Policlinico Universitario Agostino Gemelli, IRCCS, Rome, Italy

## Abstract

Lung cancer is one of the deadliest and most common malignancies in the world, representing one of the greatest challenges in cancer treatment. Immunotherapy is rapidly changing standard treatment schedule and outcomes for patients with advanced malignancies. However, several ongoing studies are still attempting to elucidate the biomarkers that could predict treatment response as well as the new strategies to improve antitumor immune system response ameliorating immunotherapy efficacy. The complex of bacteria, fungi, and other microorganisms, termed microbiota, that live on the epithelial barriers of the host, are involved in the initiation, progression, and dissemination of cancer. The functional role of microbiota has attracted an accumulating attention recently. Indeed, it has been demonstrated that commensal microorganisms are required for the maturation, education, and function of the immune system regulating the efficacy of immunotherapy in the anticancer response. In this review, we discuss some of the major findings depicting bacteria as crucial gatekeeper for the immune response against tumor and their role as driver of immunotherapy efficacy in lung cancer with a special focus on the distinctive role of gut and lung microbiota in the efficacy of immunotherapy treatment.

## 1. Introduction

The small (SCLC) and non-small-cell lung cancer (NSCLC) (referred as lung cancer “LC” hereafter) is one of the deadliest malignancies in the world. For 2019, the American Cancer Society estimates 116,440 and 111,710 new LC cases with 24% and 23% of new deaths per year for men and women, respectively [1]. Over the past few decades, the research on genetics of LC improved the opportunity to select patients that could benefit from the most recent immune-based therapeutic strategies [2–8].

Several clinical trials established the efficacy of immunotherapy on different tumors bringing to the approval of this new therapeutic regimen. The clinical trials CheckMate 017, CheckMate 057, and Keynote 010 demonstrated that the monoclonal antibodies (mAbs) against programmed cell death-1 (PD-1) nivolumab [9] and pembrolizumab [10] significantly improved the overall survival (OS) over docetaxel in NSCLC patients after the failure of prior platinum-based chemotherapy. Similarly, the OAK trial showed that atezolizumab [11], an anti-PD-ligand 1 (PD-L1) mAb, produced a survival benefit compared with docetaxel in the same NSCLC population. In details, the anti PD-(L)1 therapy blocks the binding of PD-1 to its ligand (PDL-1) restoring the functions of “exhausted” T cells and resulting in tumor shrinkage [12]. The immunoblocking between PD-1 and activated cytotoxic T lymphocytes (CTLs), and between PD-L1 and tumor cells, has exhibited significant clinical efficacy in different types of cancer and was currently approved for treating tumors, including advanced stage of NSCLC [13]. Consistently, nivolumab and pembrolizumab showed impressive efficacy also in SCLC [14].

Actually, five monoclonal antibodies targeting immune checkpoints have been approved by the U.S. Food and Drug Administration (FDA) for cancer treatment alone or in combination with platinum-based chemotherapy [9], although ongoing study attempts to discover new predictive biomarker of treatment response as well as new strategies to improve immunotherapy efficacy, including the combination of anti-PD-(L)1 and anti-Cytotoxic T Lymphocyte Antigen 4 (CTLA-4) agents [15, 16].

Several studies demonstrated that the gut microbiome regulates the power by which immunotherapy may stimulate the anticancer immune response (reviewed in [17]).

Commensal microorganisms are required for the maturation, education, and function of the immune system. A tight and continuous interaction of immune cells with microorganisms allows learning the difference between commensal and pathogenic bacteria. Indeed, the haematopoietic and nonhaematopoietic cells of the innate immune system are strategically located at the host-microbiome interface and are rich of pattern recognition receptors (PRRs) that sense microorganism presence [18]. This relationship leads to the concept of humans as mammalian holobionts resulting from parallel coevolution of host-eukaryotic and microbe-prokaryotic elements.

The gastrointestinal tract hosts are the most abundant and diversified microbial population. The gut microbiota is composed of 10^13^ to 10^14^ microorganisms whose genome is collectively at least 100 times the human genome [19]. Moreover, behind gut epithelia, bacteria colonize other specialized epidermal surfaces like the ductal system of exocrine organs and respiratory tract.

The human respiratory tract is the main portal of entry for numerous microorganisms. Interestingly, gut and lung microbiota are connected by a complex bidirectional axis via lymphatic [20] and blood circulation, and modification of one mucosal compartment can directly impact distant mucosal site [21].

Recent high-depth metagenomic sequencing techniques have changed our understanding of the complex microbiome ecosystem enabling the identification and quantification of individual bacterial strains and the correlation between specific microbiome asset and disease status. More interesting, wide efforts are now focused on how variations in these populations may influence response to immunotherapy.

In this review, we discuss some of the major findings depicting bacteria as crucial gatekeeper for the immune response against tumor and their role as driver of immunotherapy efficacy in lung cancer.

## 2. Role of Commensal Bacteria in Cancer Response to Immunotherapy

During early life, the immune system is broadly stimulated with the first contact to microorganisms at gastrointestinal and lung barriers [22]. This primary wave of microbial exposure exerts a long-lasting effect on immune cell function [23].

Increasing evidence supports the idea of a dynamic interaction between immune cells, microbiota, and tumor microenvironment. Gene expression analysis of tumors from antibiotic-treated mice showed a downregulation of genes related to inflammation, phagocytosis, antigen presentation, and adaptive immune response. Moreover, microbiota disruption impairs the efficacy of CpG-oligonucleotide immunotherapy affecting myeloid-derived cell functions in the tumor microenvironment [24].

Furthermore, it has been demonstrated that oral administration of *Bifidobacterium* improves response to anti-PD-L1 antibody in mouse models of cancer by inducing dendritic cell function and increasing CD8^+^ T cell accumulation in the tumor microenvironment [25]. Microbiota composition has also a key role in the immunostimulatory effects of Cytotoxic T-Lymphocyte Antigen 4 (CTLA-4) blockade. In details, Bacteroides species affect interleukin- (IL-) 12-dependent Th1 immune response facilitating tumor control in mice and patients [26].

A recent study analyzed baseline stool samples from 42 metastatic melanoma patients before immunotherapy treatment demonstrating an abundance of *Bifidobacterium longum*, *Collinsella aerofaciens*, and *Enterococcus faecium* in responding patients. Fecal transplantation of germ-free mice with stool from responding patients improved efficacy of anti-PD-L1 therapy increasing immune-mediated tumor control through the induction of T cell response [27].

The different microbiota composition between cancer patients and healthy individuals not only demonstrated diagnostic and prognostic potentials of special microbial pathogens in cancer but also suggested the idea that the manipulation of the microbiota could be a valid approach for a better therapeutic response, acting on drug efficacy or enhancing the immune system (discussed below).

The fecal microbiota transplantation (FMT) (i.e., the transfer of fecal bacteria from a donor into a recipient) that has been applied to clinical practice for the treatment of Clostridium difficile infection [28], ulcerative colitis [29–31], and irritable bowel syndrome [32] demonstrated an effect also on the systemic immune response and particularly on the mechanisms of immune surveillance against LC (Routy et al.). Routy and colleagues demonstrated that a specific host gut microbiota might contribute to patient immunotherapy response. Antibiotic-induced alterations of gut microbiota during immunotherapy treatment dampens patient response to the therapy. Interestingly, the FMT from patients sensitive to immunotherapy is able to revert the immunotherapy response in treatment-resistant patients. These findings lead to intriguing hypothesis that the modification of gut microbiota through FMT could enhance the response also in tumors resistant to immunotherapy.

The overall results of these studies open the avenue to propose a multiparameter prediction model integrating conventional parameters, such as tumor genetic alterations, with microbiota assessment to select patients most likely to respond to immunotherapies.

## 3. Effects of Gut Microbiota on LC

The role of the human gut microbiome is being increasingly accepted. From 2015 to present, more than 158 papers on high-impact journals were published and several research groups indicated the role of the gut microbiome in different diseases with a particular emphasis on cancers (https://www.ncbi.nlm.nih.gov/pubmed?term=(LUNG%20CANCER%20MICROBIOME)%20AND%20(%222015%2F01%2F01%22%5BDate%20-%20Publication%5D%20%3A%20%223000%22%5BDate%20-%20Publication%5D).

More than 100 trillion bacteria colonize the human intestines [33]. The crosstalk between the gut microbiota and the immune system contributes to the health status of the host. The application of this concept in oncology field is particularly important, and several recent papers highlighted the role of gut microbiota as one of the regulatory factors affecting both the tumor proliferation and the immunological environment of cancer, determining thus the efficacy of the treatment with the immune checkpoint inhibitors. The specific role of gut microbiota in supporting cancer development and growth is yet unclear. However, there are compelling evidences of the gut microbiota role in modulating both innate and adaptive immune response and how this influences tumor growth and immune escape [34]. Moreover, the gut microbiota is able to regulate host immunity both locally and at distal sites [35] modulating the expansion and differentiation of T cell populations. Briefly, the pathogen-associated molecular patterns (PAMPs) of the microorganisms in the intestines are recognized by the Toll-like-receptors (TLRs) on the membrane of intestinal epithelial cells. The activation of TLRs leads to the activation of signal cascade that finally results into the stimulation of immunological cells in the lamina propria. Dendritic cells and macrophages, activated in mesenteric lymph nodes (MLN), prime the naïve B and T cells to mature and differentiate, producing, thus, IgA. Differentiated T cells assume both profile of Th1 and/or Th17 proinflammatory cells activating additional effector cells as neutrophils or anti-inflammatory cells to control immune response [36–43]. Moreover, high diversity of gut microbiome supports M1 macrophage and Th1 lymphocyte differentiation, activation of helper/cytotoxic T cell, and upregulation of PD-1 expression on lymphocytes [44].

All these studies highlighted the potential of gut microbiota manipulation in cancer treatment, especially in tumors where the immunotherapy is currently adopted in clinical practice such as the LC and melanoma.

In melanoma, PD-1 inhibitors produce long-lasting responses in 30-40 percent of patients. However, these drugs do not work in the other 60-70 percent of melanoma patients for a multitude of reasons, including not having the right microbes in the gut—a condition termed “intestinal dysbiosis.” Likewise, several phase III LC clinical trials revealed that immunoblockade treatment leads to only approximately 20% of patients' overall objective response (OOR) and that median duration of response is significantly heterogeneous [45–47]. Recent studies demonstrated that gut microbiota could modulate immunotherapy response. Indeed, gut commensals such as *B. thetaiotaomicron* or *B. fragilis* are predictive factors for anti-CTLA-4 treatment in a mouse melanoma model [26].

It is therefore desirable to identify patients who would benefit more from immunotherapy and to understand what drives resistance in the patients who do not respond.

A study by Routy et al. proved that the gut microbiota plays a critical role in the response to PD-1 blockade and may have a prognostic value in LC. Moreover, the gut microbiota of patients who respond to immunotherapy with checkpoint inhibitors was different from those who do not. In particular, the authors identified an increased level of *Akkermansia muciniphila* (*A. muciniphila*) in patients who experienced longer survival. They demonstrated that gut microbiota not only was a predictor of response but also regulated the efficacy of anti-PD1 in murine models. In fact, the fecal microbiota transplantation from responder mice restored PD-1 blockade sensibility in the same models. Interestingly, the authors demonstrated that gut microbiome, and in particular *A. muciniphila*, influences efficacy of PD-1-based immunotherapy against epithelial tumors increasing the presence of tumor-infiltrated CCR9^+^CXCR3^+^CD4^+^ T cells through a IL-12-dependent signaling pathway [48].

A recent paper using data from 37 advanced NSCLC patients receiving nivolumab enrolled in the study from the clinical trials CheckMate 078 (NCT02613507) and CheckMate 870 (NCT03195491) demonstrated a strong correlation between the level of gut microbiome diversity and anti-PD-1 efficacy in advanced NSCLC Chinese patients. The patients with high gut microbiome diversity (reported as favorable gut microbiome) exhibited an increase of memory T and NK cell signatures in the peripheral blood samples. These findings provide important implications for the prediction of anti-PD-1 immunotherapy response in Chinese population with NSCLC [49].

To date, a single study examined the association among antibiotics and efficacy of immune checkpoint inhibitors. In this retrospective analysis of the data from 90 NSCLC patients treated (13 patients) or untreated (77 patients) with antibiotics prior to nivolumab therapy as second or later line of therapy, the authors demonstrated that antibiotic treatment reduced significantly both Progression-Free Survival (PFS) and OS. Although, in multivariate analysis, no statistically significant association was found between survival and prior antibiotic use, a trend concerning the negative influence of antibiotic use was conveyed. These data, although need further validations, confirmed that gut microbiota could have an important role in shaping systemic immune responses [50].

Botticelli and colleagues demonstrated that a specific gut microbiome may influence the response to immunotherapy. In particular, by using the NGS technique, the authors showed that there are higher levels of Rikenellaceae, Prevotella, Streptococcus, Lactobacillus, Bacteroides plebeius, Oscillospira, and Enterobacteriaceae in the stool of NSCLC patients than in healthy controls. Moreover, patients who respond to nivolumab treatment had less abundance of *Ruminococcus bromii*, *Dialister*, and *Sutterella* spp. than not responders [51].

The concept of immunomodulatory ability is also applicable to the chemotherapy regimen able to regulate the immune system. Cyclophosphamide is well known for its antineoplastic and immunomodulating ability and was registered for early and advanced breast cancer. In a transgenic tumor mouse model of autochthonous lung carcinogenesis, this alkylating agent alters the composition of microbiota in the small intestine inducing translocation of specific Gram-positive bacteria, including *Lactobacillus johnsonii* (growing in >40% cases), *Lactobacillus murinus*, and *Enterococcus hirae*, into secondary lymphoid organs [52]. Here, the Gram-positive bacteria stimulate the generation of a specific subset of “pathogenic” T helper 17 (pTh17) cells and memory Th1 immune response. In germ-free or antibiotic-treated animal models, the absence of these bacteria leads to a reduction in pTh17 response and cyclophosphamide tumor resistance. Adoptive transfer of pTh17 cells partially restored the antitumor efficacy of cyclophosphamide. These results suggest that the gut microbiota helps shape the anticancer immune response in LC patients [53].

## 4. Effects of Lung Microbiota on LC

The lung is constantly exposed to microorganisms from the air and the upper respiratory tract; therefore, it is not a “sterile place” as previously believed. Acquisition of lung microbiome is a crucial event in newborn to protect the lung from injuries [54]. Lung tissue hosts a unique microbiome asset with less diversity, compared to the intestinal one, but equally affected by drugs, disease, and eating habits, which can create a selective pressure on reproducing communities. The specific composition of the lung microbiome results from the balance of three phenomena: microbial immigration, microbial elimination, and the relative reproduction rates of its members [55].

Dysbiosis of lung microbiome ecosystem and the epithelial integrity loss in heavy smokers could be the initial cause of inflammation in chronic obstructive pulmonary disease and LC [56]. A comparative analysis of 142 LC patients and 33 healthy controls reveals a distinct lung microbiome profile associated with tumor tissue [57]. Moreover, epidemiological evidence indicates a significant association between prolonged antibiotic exposure and incidence of LC [58].

Exacerbations of chronic lung disease have shown correlation with microbiota disorder of the respiratory tract. Respiratory dysbiosis is closely linked to a dysregulated host immune system, which in turn further affects lung microenvironment promoting inflammation [59].

On the other hand, a recent study claims that depletion of local commensal microbiota or blockade of the downstream cellular/molecular immune mediators suppresses the development of lung adenocarcinoma. By using the conditionally genetically engineered mouse model (GEMM) of lung adenocarcinoma, the authors demonstrated that commensal bacteria stimulate production of IL-1*β* and IL-23 from myeloid cells via a Myd88-dependent pathway. This event leads to proliferation and activation of tissue resident *γδ* T cells with a consequent increased production of effector molecules, such as IL-17, to promote inflammation and tumor cell proliferation [60]. However, this study does not deep investigate the specific strain composition of the lung microbiota responsible for lung tumor development.

Many efforts have been focused on the discovery of bacterial diagnostic biomarkers for LC [61, 62].

These biomarker discovery studies often used saliva, sputum, bronchoscopic samples, or bronchoalveolar lavage fluid instead of direct lung biopsy, which is not performed on healthy subjects. However, lung tissue remains the most accurate sample to study lung microbiome alternations [63]. A study evaluating saliva microbiota revealed that bacterial profiles are significantly altered in LC patients compared to those from control subjects. In particular, *Capnocytophaga*, *Selenomonas*, and *Veillonella* were found to be more abundant in both lung squamous cell carcinoma and adenocarcinoma patients whereas *Neisseria* was less abundant than in the controls [64].

Another study compared bronchial brushing samples from cancerous site and contralateral noncancerous site of 24 LC patients and 18 healthy controls. The authors demonstrated that LC-associated microbiota profile is extremely divergent from that found in healthy subjects with a significant decrease in microbial diversity. More interestingly, the alterations of microbiota composition in unilateral lobe LC patients are extended to the contralateral noncancerous site suggesting a deep change of the whole lung microenvironment, which is linked to the development of LC [65].

Although increasing evidence has highlighted the key role of commensal microbiota in tumor-immune system interaction and treatment response, the main efforts have been focused on gut microbiota. Less is known on how lung microbiota could affect antitumor immunity and immunotherapy response.

Evidence suggests that manipulation of the composition of local flora may influence the ability of the host to generate an immune response that could mount both local and distal antitumor protective responses ameliorating the efficacy of immunotherapy treatment.

To date, several interesting clinical trials are attempted to study the role of lung microbiota on the efficacy of immunotherapy-based treatment in LC (Table 1).

An ongoing observational clinical trial (NCT03688347) at Iowa Institute of Human Genetics (Iowa, US) is currently recruiting patients with advanced or recurrent LC (and other solid tumors) that initiate a new line of immunotherapy, either alone or in combination with chemotherapy, targeted therapy, or other immunotherapy agents.

Recently, Stevenson et al. isolated and identified *Enterococcus gallinarum* MRx0518, a commensal Gram-positive species, demonstrating the antitumor efficacy of this bacteria strain in mouse models of different solid tumors, including LC. MRx0518, and more specifically its flagellin, acts on both the innate and the adaptive immune system showing strong immunostimulating properties. Its inactivation resulted in complete abrogation of the TLR5-mediated activation of NF-*κ*B [66, 67].

Based on these exciting results, the NCT03934827, a single center, open label clinical trial, is aimed at studying MRx0518 in combination with pembrolizumab in patients with LC and other solid tumors (at MD Anderson Cancer Center Houston, Texas, US). This study will assess the safety and tolerability and clinical benefit of MRx0518 in combination with pembrolizumab through the collection of adverse events.

Moreover, the NCT03168464 Interventional Clinical Trial at Weill Medical College of Cornell University (New York, US) is aimed at evaluating the association of ORR with changes in the microbiome in NSCLC patients with metastatic disease who have failed at least one prior treatment.

Although these studies are still in their infancy, they will provide a valid contribution in the exact determination of the role of the local microbiota in the response to immunotherapeutic agents and, on the other hand, will provide both new prognostic biomarkers and a powerful alternative tool to modulate the patient outcome.

## 5. Gut-Lung Microbiota Axis

**Figure 1:**
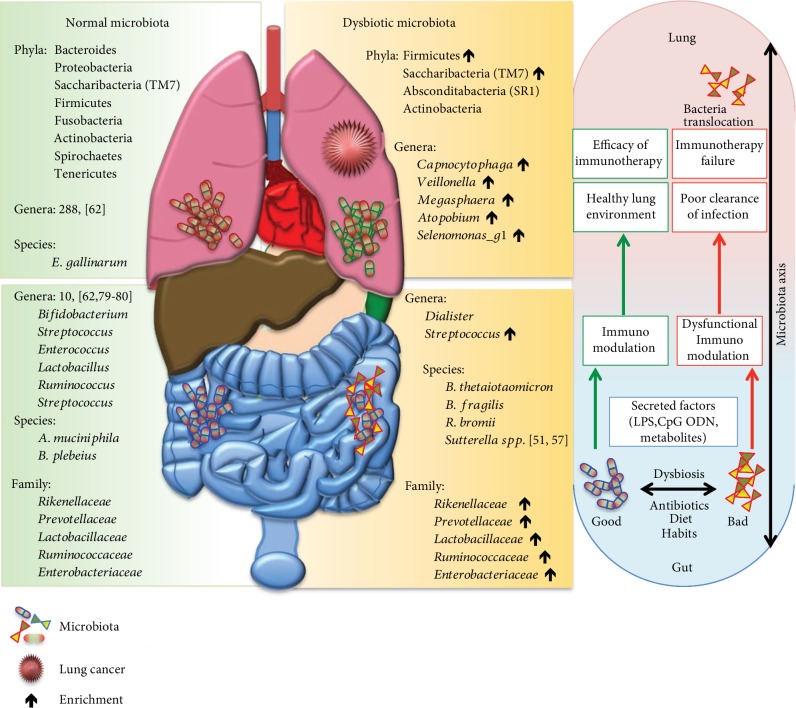
Increasing evidence supports the idea of a dynamic influence between host and microbiota. The fine line between human health and disease can be driven by friend (green) or foe (red) microbiota. We reported the main bacteria that could be responsible for the transition from a health to a pathological status. Commensal microorganisms are required for the maturation, education, and function of the immune system. A tight and continuous interaction of immune cells with microorganisms allows learning the difference between commensal and pathogenic bacteria that could influence immunotherapeutic treatment.

The interaction between gut microbiota and host cells in the intestinal mucosa occurs in several ways. The pathogen-associated molecular patterns (PAMPs), provided by gut microbiota, serve as ligands for different Toll-like receptors (TLRs) on the surface of the intestinal epithelial cells (IECs). PAMPs from different microbiomal origin, such as lipopolysaccharide (LPS) or CpG ODN from bacteria, or viral double-stranded RNA, or toxin from parasites and fungi could activate TLR innate-adaptive immunity [68, 69] (Figure 1). In a similar way, also lipoteichoic acid (LTA), the main component of the Gram-positive cellular wall seems to function as potent immune activator with a signaling similar to the LPS activation pathway.

Indeed, the immune system through plasma cells and IgA secretion into the lumen of the gut could regulate in turn microbiota population [70]. Moreover, commensal bacteria and their metabolites (i.e., short-chain fatty acids (SCFAs) like butyrate, propionate, and acetate) directly stimulate IECs regulating immune cells. SCFAs might regulate the immune system through regulation of G-protein-coupled receptors (GPRs) and histone deacetylase [71], modulating epithelial and immune cell functions. Other cell types have also emerged as targets of SCFAs, including monocytes, dendritic cells, T cells, and intestinal epithelial cells [72].

In dendritic cells, treatment with SCFA butyrate is associated with decreased expression of the proinflammatory cytokines IL-12 and IFN-*γ* and increased expression of Th2 cytokines [72]. Some evidence suggests that butyrate may regulate the ability of dendritic cells to present antigen and to prime T cells [73].

The gastrointestinal and respiratory tracts, although physically distant organs, are part of a shared mucosal immune ecosystem named the gut-lung axis [74]. Gut microbiota dysbiosis has been implicated in several lung diseases. Indeed, restoring microbiota in the gut of mice resulted in reduced severity of pneumonia [75].

It has been hypothesized a bidirectional crosstalk between the two microbiota entities which means that alteration of one compartment could impact on the other one.

This concept opens the possibility to indirectly modify lung bacterial composition, which represents the population physically close to lung tumor microenvironment, through gut microbiota modification strategies, such as fecal transplantation.

The dynamic crosstalk between the two compartments occurs through a direct translocation of bacteria from one to the other site or through the release into the bloodstream and the lymphatic system of bacteria-derived immunomodulatory molecules, which affect systemic immunity [75–80].

The massive crosstalk between the microbiota of gut-lung axis and its decisive role in inflammation and against lung infections could open to new therapeutic and immunization strategies.

## 6. Conclusions

The straight interaction between microbiota and host epithelial barrier is required for the maturation, education, and function of the immune system impacting the host's health but also the power of immunotherapy to boost anticancer response. The molecular crosstalk between the gut and lung microbiota and anticancer immune regulation represents a novel area of research. Potentially, the microbiota could modulate and eventually potentiate an immune response by the release of proinflammatory cytokines, metabolites, or nucleic acids, allowing a microbiota-based selection of patients who could benefit from specific immunotherapy treatment.

However, microbiota composition differs widely according to host genetics and racial characteristic as well as diet and eating habits. These variables are closely related to geographical location, suggesting therefore the need of more in-depth clinical research studies, looking at ethnic diversity as well as eating habits and environment-related factors.

These substantial divergences in the basal microbiome components of different study populations question the universality of the microbiome-based findings and recommend taking into consideration more geographically tailored approaches [81]. Because this research area is still in its infancy, new efforts are necessary to determine the role of the microbiota in the response to immunotherapeutic agents and also to comprehensively illustrate the gut-lung axis and its implications.

## Figures and Tables

**Table 1:** Clinical trials investigating the role of microbiota in lung cancer patients receiving immunotherapy.

ClinicalTrial.gov identifier	Title	Conditions	Study type	Intervention/treatment	Estimated enrollment (patients)	Primary outcome	Secondary outcome
NCT03688347	Microbiome in lung cancer and other malignancies	Lung cancer and other solid tumors	*Observational*	Nasal, skin, and oral swab, stool collection, and microbiota analysis	40	Identify and compare bacteria within given samples through a standard protocol and 16S rRNA amplicon; correlate data from samples with patient clinical information regarding overall response rates	Correlate data from samples with patient clinical information regarding overall response rates

NCT03934827	MRx0518 in patients with solid tumours waiting surgical removal of the tumour	Lung cancer and other solid tumors	*Phase 1*	MRx0518 *vs.* placebo capsules	120	Safety and tolerability of MRx0518 as determined through the collection of the number and severity of AEs, SAEs, changes in biochemistry, haematology, urinalysis laboratory results, and vital signs	Response of MRx0518 determined by the measurement of tumor markers; OS of patients who receive MRx0518 compared to placebo

NCT03168464	Radiation and immune checkpoints blockade in metastatic NSCLC (BMS # CA209-632)	Metastatic NSCLC	*Phase 1, 2*	Nivolumab, ipilimumab, and radiation therapy	45	Enhance ORR to the combination of nivolumab/ipilimumab in chemorefractory NSCLC and double the ORR of ipilimumab/RT, from 18% based on intent to treat to 36%	Changes in TCR repertoire in peripheral blood are associated with response to treatment; serum markers IFN-b, CXCL11, sMICA, sMICB levels/changes associated with patients' response to the treatment; PFS; OS; associations of ORR with changes in the microbiome

*vs.*: versus; AEs: adverse events; SAEs: serious adverse events; OS: overall survival; NSCLC: non-small-cell lung cancer; ORR: overall response rate; RT: radiotherapy; TCR: T cell receptor; PFS: progression-free survival.
